# Sublethal Caspase Activation Promotes Generation of Cardiomyocytes from Embryonic Stem Cells

**DOI:** 10.1371/journal.pone.0120176

**Published:** 2015-03-12

**Authors:** Ivana Bulatovic, Cristian Ibarra, Cecilia Österholm, Heng Wang, Antonio Beltrán-Rodríguez, Manuel Varas-Godoy, Agneta Månsson-Broberg, Per Uhlén, András Simon, Karl-Henrik Grinnemo

**Affiliations:** 1 Division of Cardiothoracic Surgery and Anesthesiology, Department of Molecular Medicine and Surgery, Karolinska Institutet, Karolinska University Hospital, Stockholm, Sweden; 2 Department of Medical Biochemistry and Biophysics, Karolinska Institutet, Stockholm, Sweden; 3 Department of Cellular and Molecular Biology, Karolinska Institutet, Stockholm, Sweden; 4 Department of Clinical Science, Intervention and Technology, Karolinska Institutet, Stockholm, Sweden; 5 Division of Cardiology, Department of Medicine Huddinge, Karolinska Institutet, Karolinska University Hospital, Stockholm, Sweden; Georgia Regents University, UNITED STATES

## Abstract

Generation of new cardiomyocytes is critical for cardiac repair following myocardial injury, but which kind of stimuli is most important for cardiomyocyte regeneration is still unclear. Here we explore if apoptotic stimuli, manifested through caspase activation, influences cardiac progenitor up-regulation and cardiomyocyte differentiation. Using mouse embryonic stem cells as a cellular model, we show that sublethal activation of caspases increases the yield of cardiomyocytes while concurrently promoting the proliferation and differentiation of c-Kit^+^/α-actinin^low ^cardiac progenitor cells. A broad-spectrum caspase inhibitor blocked these effects. In addition, the caspase inhibitor reversed the mRNA expression of genes expressed in cardiomyocytes and their precursors. Our study demonstrates that sublethal caspase-activation has an important role in cardiomyocyte differentiation and may have significant implications for promoting cardiac regeneration after myocardial injury involving exogenous or endogenous cell sources.

## Introduction

During the last decade a number of studies have reformed the classic view of the heart as a post-mitotic organ. It has been demonstrated that mammalian cardiomyocytes retain the capacity for cell division throughout their lifespan [1,2] and endogenous cardiac progenitor cells have been identified in the adult heart [3,4,5]. Stem cells expressing the growth factor receptor c-Kit [3] and LIM homeodomain transcription factor Islet-1[4,6] have been suggested as the main cardiac progenitor populations in the heart. Whether these cells have a role in physiological cardiomyocyte turnover and stress-induced cardiac repair remains largely disputed, even if experimental studies suggest their activation by ischemia-reperfusion, myocardial infarction, pregnancy, chronic heart failure and diffuse acute myocardial damage [5,7,8,9]. The mechanism by which heart injury modulates the activation of cardiac stem cells is still poorly understood, where several studies suggest a role of released paracrine factors from cardiomyocytes, changes in the metabolic environment, exosome signaling, pro-inflammatory cytokines as well as recruitment of bone-marrow mesenchymal stem cells [10]. Still, an unresolved question is whether different types of injuries with diverse effects on the heart, can all converge into a common cardiac progenitor cell response mediated through a common stress-activated signaling pathway [11,12].

Caspases, a family of cysteinyl-aspartate-specific proteases, are master regulators of apoptosis and their activation is considered mandatory to initiate and execute programmed cell death [13,14]. However, recent findings suggest a number of non-apoptotic roles of caspases like self-renewal and differentiation of embryonic stem cells [15], hematopoietic stem cells [16] as well as differentiation of skeletal muscle and neurons [17,18]. Experimental activation of these proteases might be used for unraveling cell fate decisions beyond programmed cell death.

We describe that sublethal apoptotic stimuli modulate embryonic stem cells (ESC) differentiation into cardiomyocytes through a caspase-dependent mechanism. Our findings suggest that caspase activation promotes cardiomyocyte differentiation through the activation of c-Kit^+^/α-actinin^low^ cardiac progenitor cells. This indicates a direct link between caspase activation and initiation of the cardiogenic programs in undifferentiated cells, inducing formation of cardiac progenitors and cardiomyocytes.

## Materials and Methods

### Embryonic Stem Cell Culture and Differentiation

Murine embryonic stem cells (mESC-line CGR8) were kindly provided by Prof. Richard Lee, Harvard Medical School [19]. The mESCs were stably transfected with the cardiac-specific α-myosin heavy chain (α-MHC) promoter tagged with green fluorescent protein (GFP) and thus cardiomyocyte differentiation could be readily assessed via observation of GFP [19]. The cells were propagated in Glasgow Minimum Essential Medium (GMEM) (Invitrogen) supplemented with 1,000 U/ml Leukemia Inhibitory Factor (LIF, ESGRO, Millipore), 1mM Sodium Pyruvate, 1x Non-Essential Amino Acid, 15% Knockout Serum Replacement (Invitrogen), 10^-4^M β-mercaptoethanol, and 1 x Penicillin-Streptomycin. Cells were maintained in Petri dishes coated with 0.1% gelatin. Differentiation was carried out as hanging drops in differentiation medium (10% FBS without LIF) in which embryoid bodies (EBs) were formed in 2 days (D0–2). The EBs were subsequently incubated for 2 days (D2–4) in suspension followed by 17 days (D5–21) on gelatin-coated coverslips in 24-well culture plates.

### Apoptosis Induction and Caspase Inhibition

The EBs were incubated with 1nM-10μM Staurosporine (STS, Sigma, cat# S6942, reconstituted in DMSO) or with DMSO as a negative control added to the medium 5 h prior to the measurement of caspase activation. The sublethal dose of STS used in all experiment was 100 nM. To examine the effects of caspase inhibition, EBs were incubated with 10 μM of the pan-caspase inhibitor Q-VD-OPhydrate (Sigma, cat#SML0063). All experiments were performed in triplicate.

### RNA Extraction and Quantitative Real-Time Polymerase Chain Reaction (RT-PCR)

Total RNA was extracted using the PicoPure RNA Isolation Kit cat# 12204–01 (Arcturus, Applied Biosystems). RNA concentrations and purities (A260/280) were measured in a NanoDrop spectrophotometer (ND-1000) (Nanodrop technologies, Wilmington, DE, USA).

Two micrograms of total RNA was reverse-transcribed by High Capacity cDNA Reverse Transcription cat# 4368814 (Applied Biosystems) in a total volume of 20 μl.

After reverse transcription, 20 ng cDNA was used for real-time quantitative PCR, performed with a 7500 Fast real-time PCR system (Applied Biosystems Inc., Foster City, CA, USA) using the following TaqMan Gene Expression Assays (Applied Biosystems) Isl1 Mm 00517585_m1, Kit Mm 004452212_m1, Myh6 Mm00440359_m1, GATA4 Mm00484689_m1, Nkx 2.5 Mm00657783_ m1, Tnnt2 Mm01290255_m1, Pou5f1 Mm03053917_g1, B2m Mm00437762_m1. β-2-microglobulin was used as an endogenous control to correct for potential variations in cDNA loading.

PCR reactions were performed in 96-well MicroAmp Fast Optical plates cat# 4346906 (Applied Biosystems). Amplification reagents (20 μl) contained 4 μl sample in TaqMan Universal PCR Mastermix. Relative mRNA levels are expressed as means ± SE of fold change relative to control. Data are representative of three independent experiments.

### Immunofluorescence Staining

Embryoid bodies were grown on coverslips in 24 well plates (1 EB per coverslip). The coverslips were fixed in ice-cold methanol for 10 minutes, blocked with 5% of the relevant serum for one hour followed by overnight incubation with primary antibodies: α-actinin (Mouse mAb, Sigma), c-kit (D13A2 Rabbit mAb, Cell Signaling), active caspase 3 (Rabbit pAb, Cell Signaling) and active caspase 9 (Rabbit pAb, Cell Signaling). Subsequently, the coverslips were incubated with Alexa Fluor 488-labeled goat-anti-mouse and Alexa Fluor 546-labeled goat-anti-rabbit secondary antibodies (Invitrogen, Carlsbad, CA). EBs were visualized in an inverted confocal microscope (Olympus Optical Co. Ltd., Tokyo, Japan). Data from three independent experiments are presented. Global colocalization analysis was performed by calculating the overlap coefficient using ImageJ Colocalization Finder plug-in software. For the quantitation of colocalizing pixels we calculated effective colocalization coefficients based on Manders local coefficients *M1* and *M2*, as previously described [20].

### Continuous Visualization of Live Cells

Prospective visualization of GFP expression levels was done using CELL-IQ SLF continuous live cell imaging and analysis system platform (CM Technologies, Tampere, Finland). EBs growing in 24-well dishes were stimulated as indicated and then placed into the CELL-IQ imaging system. During the imaging period, conditions were maintained at 37°C, 5% CO_2_. A whole-well fluorescence confocal z-stack composed of 6x6 images was taken for each well for every 60 minutes, during a total recording time of 72 h. The culture medium was replaced every second day. Single cell tracking analysis was performed off-line using CELL-IQ analysis software.

### EdU Treatment and Staining

Click-iT EdU Alexa Fluor 647 Flow Cytometry Assay Kit (Molecular Probes) cat# A10202 Life technologies was used. After the indicated treatments, EBs were pulsed for 30 minutes with 10 μM EdU, washed 3 times with ice-cold PBS and then fixed with methanol. EdU staining was performed according to the manufacturer’s instructions.

### In Vitro Caspase Cleavage Assay

The Caspase-Glo 3/7 kit (Promega, cat#G8090), Caspase-Glo 9 (Promega, cat#G8210), Caspase-Glo 8 (Promega, cat#G8200), Caspase 10 Fluorometric assay (Abcam, cat# ab65662), Caspase 12 Fluorometric assay (Abcam, cat#ab65664) were used according to the manufacturers´ protocols in order to directly measure the respective caspase activity. Recombinant caspase-3 (BD Bioscience, cat# 556471) was used to measure the variability of these assays. The signal was measured using FLUOstar OPTIMA (BMG LABTECH GmbH, Germany).

### Western Blot

Total protein was extracted using RIPA buffer (Sigma, cat# R0278), containing a protease inhibitor cocktail (Complete Mini, Roche, cat# 04693124001). Equal amounts of proteins were denatured in loading buffer, separated on Mini-PROTEAN TGX polyacrylamide gels (Bio-Rad, cat# 456–9034) and electro transferred into Immune-Blot PVDF membranes (Bio-Rad, cat# 162–0177). The membranes were blocked with blocking solution (Invitrogen, cat# 000105) and incubated with primary antibodies against c-Kit (rabbit monoclonal; Cell Signaling: 1:1000, cat# 3074S), followed by peroxidase-conjugated secondary antibodies (anti-rabbit IgG; Sigma: 1:10000, cat# A6667, or anti-mouse IgG; Sigma: 1:1000, cat# A4416, respectively). Amersham ECL prime western blotting detection reagent (GE Healthcare, cat# RPN2236) was used and the chemi-luminescent signal was detected using a Bio-rad ChemiDoc XRS+ (Bio-rad). Data are representative of three independent experiments.

### Mitochondrial Potential

Embryoid bodies were pre-loaded with 1μM tetramethylrhodamineethyl (TMRE, Molecular Probes) for 15 min at 37°C and images were acquired with an up-right microscope (excitation 543 nm; emission 500–600 nm). Measurements are expressed as percentage of fluorescence intensity relative to basal fluorescence. Maximum mitochondrial depolarization was obtained using the protonophore FCCP (carbonyl cyanide-p-trifluoromethoxyphenyl-hydrazon).

### Statistics

All data are presented as mean ± standard error. Independent 2 tailed t-test was employed to calculate the statistical significance between two independent groups. p<0.05 and p<0.001 were considered to be statistically significant and highly significant respectively. Statistical analyses were performed with the SPSS software version 21.0.

## Results

### Sublethal Pro-Apoptotic Stimuli Induce Caspase Activation without Substantial Mitochondrial or Cell Membrane Damage in Differentiating Embryoid Bodies

**Fig 1:**
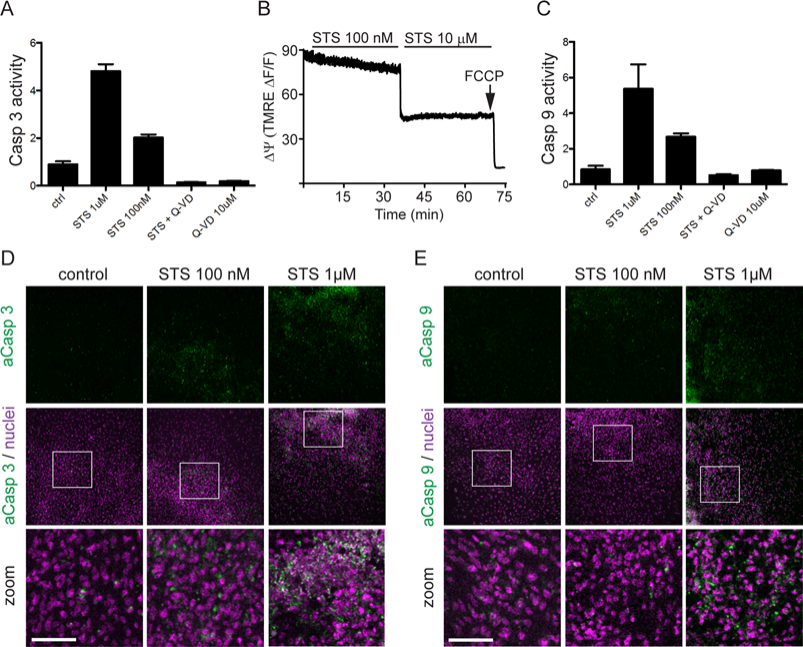
STS induces dose-dependent activation of caspases in differentiating EBs. (A and C) Enzymatic caspase 3 and 9 activity in response to stimulation with 1μM STS, 100 nM STS +/- Q-VD-OPH, and 10 μM Q-VD-OPH for 5 h. (B) Dynamic changes in mitochondrial potential detected in EBs as assayed by TMRE loss. (D and E) Caspase3 (active caspase 3 immunofluorescence) and caspase 9 (active caspase 9 immunofluorescence) activation in response to 1μM and 100 nM STS. Bars represent100 μm.

In order to test the hypothesis that sublethal activation of caspases influences cardiomyocyte differentiation, we first established a caspase activation protocol in cultures of differentiating mouse embryoid bodies (EBs). Staurosporine (STS), an ATP-competitive kinase inhibitor, was applied for 5 h in a dose-dependent manner followed by analysis of caspase activation and cell death. At micromolar concentrations of STS, massive plasma membrane permeabilization was observed in live cultures, as assessed by Propidium Iodide (PI)/Hoechst nuclear counterstaining (>40% PI positive cells at 1μM STS and > 65% PI positive cells at 10 μM STS) (S1A Fig.). In contrast, exposure to 100 nM STS resulted in less than 7% PI positive cells (S1A Fig.), while still inducing significant activation of the caspases 3 and 9 (Figs. 1A, 1C, 1D and 1E). The caspase 3 activation was dose-dependent and 100 nM STS induced a significant 1.8 fold increase compared to control (S1B and S1C Figs.). Caspase 9 demonstrated a similar response (Fig. 1C). Further optimization of the apoptotic stimulus was done by tetramethylrhodamineethyl (TMRE) analyses. TMRE is a mitochondrial membrane potential (MMP) sensitive probe, which accumulates within the polarized intact mitochondria [21]. Thus, the loss of TMRE signal is an indirect indicator of mitochondrial membrane permeabilization and depolarization, which in turn is a hallmark of apoptotic cells. Our analyses demonstrated a significant decrease in the MMP when treating the cells with μM compared to nM concentrations of STS (Fig. 1B). Based on these data, 100 nM STS was chosen as the target sublethal concentration, since it activated caspase-mediated pathways while at the same time limited the number of apoptotic cells. Caspase 8 was also activated (S1D Fig.). This activation is most probably mediated through the STS induced recruitment of Fas-associated protein with Death Domain (FADD) [22]. However, 100 nM STS did not activate caspase 10, which in turn is processed by caspase 8 leading to cleavage and activation of caspase 3. On the other hand, 1 μM STS concentration, most commonly used to induce apoptosis, lead to significant caspase 10 activation (S1E Fig.). Caspase 12, known to process inflammatory cytokines, was not activated following treatment with 100 nM STS (data not shown). The pan-caspase-inhibitor Q-VD (OMe)-OPH reversed the STS-induced caspase 3, 8 and 9 activities down to levels below the untreated control (Figs. 1A, 1C, S1C and S1D), which indicates that caspases are naturally activated and involved in the differentiation of the EBs.

### Cardiac Differentiation Is Enhanced in Response to Sublethal Pro-apoptotic Stimuli

**Fig 2:**
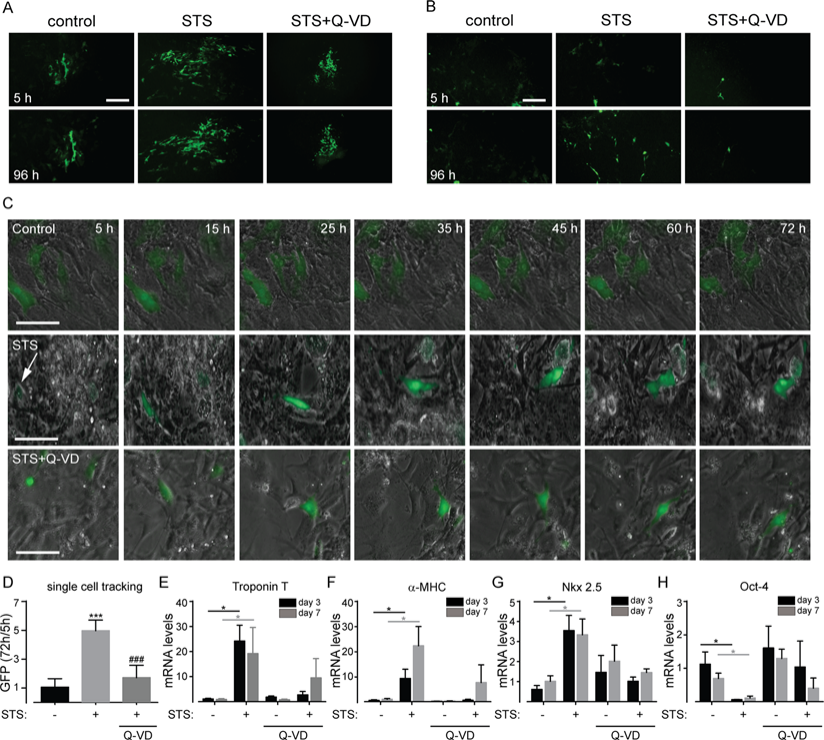
Sublethal caspase activation enhances cardiac differentiation. (A) GFP expression levels in EBs exposed to vehicle or 100 nM STS +/- Q-VD-OPH (upper panels) and then cultured for additional 96 h (lower panels). Cardiac α-MHC promoter was driving GFP expression and differentiation was indicated as increase in GFP fluorescence compared to control. (B) In GFP negative zones of the EBs, new GFP positive cells were observed upon STS treatment. (C) Prospective long-term imaging of GFP expression in EBs exposed to control vehicle, 100 nM STS or 100 nM STS + Q-VD-OPH for 5 h and GFP expression was monitored during the following 72 h. (D) GFP expression level is expressed as the ratio between GFP levels at 72 h over GFP levels at 5 h, as monitored in individual, single cells. Relative mRNA expression levels of TnT (E), α-MHC (F), Nkx 2.5 (G) and Oct-4 (H) in EBs exposed to STS +/- Q-VD-OPH and collected for analysis after 3 and 7 days. Arrow indicates an individual, single cell after 5 h exposure to STS, which is then followed for 72 h,*p<0.05 vs control, ***p <0.001 vs control, ### p<0.001 vs STS.

To test the effect of sublethal caspase activation on cardiac differentiation, we used mouse ESCs, which were stably transfected with a GFP reporter under the control of the α-myosin heavy chain (α-MHC) promoter. Upon differentiation into cardiomyocytes, these cells express GFP, which could be quantified and monitored in fixed cells and also in live cultures. EBs were cultured in differentiation medium for three weeks followed by 5 h incubation with 100 nM STS followed by a recovery period ranging from 24 h to 7 days. After a 96 h recovery period, we observed an increase in the proportion of GFP^+^ cells in STS treated EBs (Fig. 2A). STS-treatment generated GFP^+^ cells together with beating clusters of cells displaying low GFP expression (Fig. 2B). Pre-incubation of EBs with Q-VD-OPH prevented the effects induced by STS (Figs. 2A and 2B). These results suggest that sublethal caspase activation promotes cardiac differentiation. To further test this hypothesis, we continuously monitored GFP levels for 3 days after STS treatment using long-term live cell imaging. In control cells, GFP levels remained unaltered, while STS treatment significantly increased the GFP levels (Figs. 2C and 2D). Notably, pre-incubation with Q-VD-OPH inhibited this effect (Figs. 2C and 2D), suggesting that STS promoted cardiac differentiation in a caspase-dependent manner. This conclusion was further supported by the gene expression analysis of cardiac differentiation markers in response to STS treatment. TroponinT (Fig. 2E), α-MHC (Fig. 2F), and Nkx 2.5 (Fig. 2G) mRNA expression levels were significantly up-regulated in STS-treated cells after 3 and 7 days, whereas the expression of Oct-4 (Fig. 2H), a hallmark of pluripotency, was down-regulated. Notably, all these effects were again inhibited by Q-VD-OPH pretreatment. Altogether, these results indicate that sublethal caspase activation promotes differentiation of mESCs into cardiomyocytes.

### Caspase Activation Promotes Expansion and Differentiation of c-Kit/α-actinin^low^ Progenitor Cells

Others and we have reported that the cardiac progenitor cell population expressing c-Kit is responsive to various stressful stimuli like ischemia-reperfusion, myocardial infarction, and acute diffuse myocardial damage [5,8,23]. To address these findings in the context of our study, EBs in the beginning of their differentiation were treated for 5 h with 100 nM STS and subsequently cultured for three weeks. We observed a significant increase in c-Kit level suggesting that sublethal caspase activation in mESCs under undifferentiated conditions up-regulates c-Kit expression (S2A Fig.).

However, the differences in levels of α-actinin and troponin T were not significant (data not shown), suggesting that apoptotic stimuli may preferably promote differentiation of cardiac progenitors and early cardiomyocytes.

**Fig 3:**
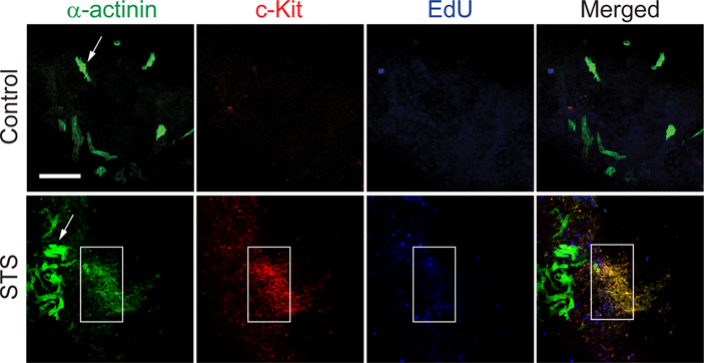
Sublethal caspase activation promotes c-Kit + cardiac progenitors proliferation. Counterstaining for α-actinin and c-Kit in EBs exposed to 100 nM STS for 5 h and then cultured for additional 7 days. Area defined by the rectangle indicates clusters of c-Kit/α-actinin^low^ cells, and arrow indicates α-actinin^high^ cardiomyocytes. 7 days after the indicated treatments, EBs were pulsed for 30 minutes with EdU and then stained for α-actinin (green), c-Kit (red) and EdU (blue). Blue nuclei indicate proliferating cells. Bars represent 100 μm.

When EBs three weeks after differentiation were treated with STS, the c-Kit positive progenitors not only proliferated, they also differentiated into cells expressing α-actinin (Fig. 3). Interestingly, c-Kit staining did not co-localize with mature strongly α-actinin positive cells, arguing against c-Kit re-expression in terminally differentiated cardiomyocytes (Fig. 3). Indeed two population of α-actinin positive cells were observed: first; cells which strongly stained for α-actinin with elongated morphology, resembling more differentiated cardiomyocytes (α-actinin^high^ cells) and secondly; more immature cells with round morphology and weak α-actinin staining, (α-actinin^low^ cells). Notably, we observed that the second population with immature α-actinin^low^ cells co-localized with cells expressing c-Kit, forming clusters in the proximity of the more differentiated, α-actinin^high^ cells (Fig. 3). This observation supports the hypothesis that cardiomyocytes were generated *de novo* from pre-existing cardiac progenitor cells in a caspase-dependent manner. The hypothesis was further supported by the fact that STS treatment increased incorporation of the nucleotide analogue 5-ethynyl-2´-deoxyuridine (EdU) in c-Kit^+^/ α-actinin^low^ cells without affecting the population of mature, α-actinin^high^ cells (Figs. 3 and S2B).

### Caspase Activation Affects Progenitor Cells and Immature Cardiomyocytes

**Fig 4:**
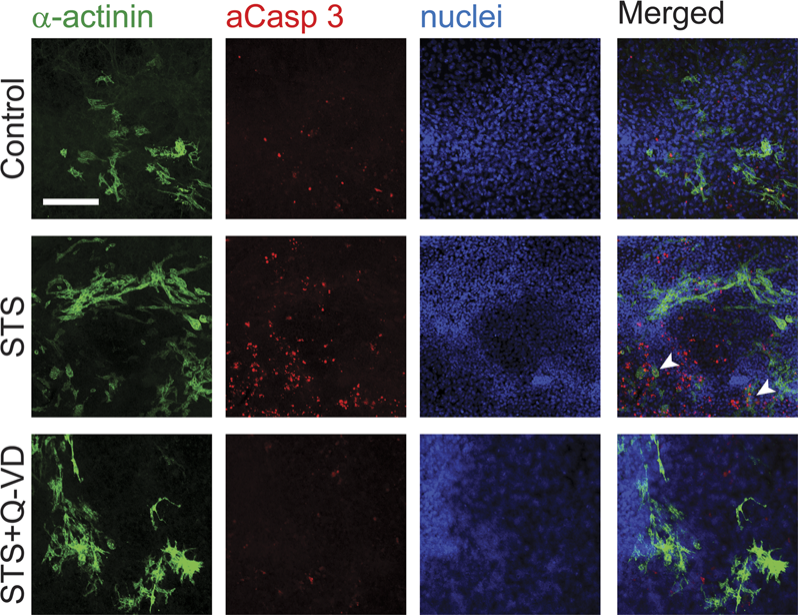
Caspase activation occurs mostly in non-differentiated cardiomyocytes. (A) Counterstaining of α-actinin (green), active caspase 3 (red) and nuclei (Dapi, blue) in EBs exposed to control vehicle or 100 nM STS +/- Q-VD-OPH for 5 h as indicated. Arrowheads indicate α-actinin^low^ cells positive for active caspase 3.

To further identify the cell population that respond to STS treatment, differentiated EBs were exposed to the sublethal stimulation protocol and stained for α-actinin and active caspase 3 (Fig. 4). In EBs subjected to control treatment, a low proportion of cells stained positive for active caspase 3. As expected, STS-treatment substantially increased the total number of active caspase 3 positive cells, which was mainly detected in α-actinin negative cells. The more mature α-actinin^high^ cardiomyocytes rarely co-stained for active caspase 3. Importantly, α-actinin^low^ cells were also positive for active caspase 3 (Fig. 4 arrowheads), suggesting that caspase 3 is activated in undifferentiated progenitor cells, but not in mature cardiomyocytes. We also evaluated the expression of cardiac and pluripotency markers directly after STS-treatment and found that the expression levels of troponin T, α-MHC, Nkx 2.5 and Oct-4 were not significantly altered (data not shown), arguing against the possibility that a particular cell population had expanded due to a positive selective pressure. Furthermore, hypertrophy of pre-existing cardiomyocytes was unlikely to account for the observed STS-induced effects, since specific hypertrophy markers, β-MHC and atrial natriuretic peptide (ANP), were not affected by the STS treatment (S3 Fig.).

Altogether, these results are consistent with caspase activation as a critical stimulus for activation, proliferation and differentiation of cardiac progenitor cells.

### Caspase Inhibition Suppresses Cardiac Differentiation

**Fig 5:**
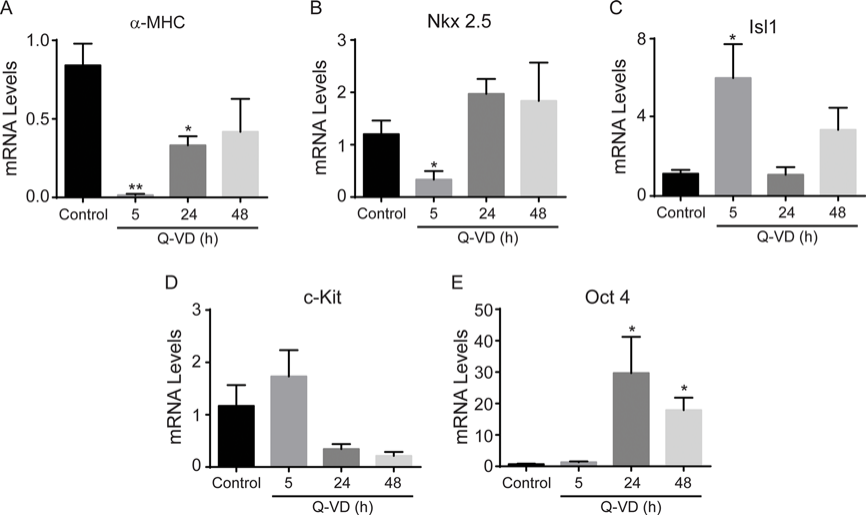
Opposing effects of caspase inhibition on cardiac progenitor cell and differentiation markers expression. Relative mRNA levels of α-MHC (A), Nkx 2.5 (B), Isl1 (C), c-Kit (D) and Oct-4 (E) in EBs exposed to control vehicle or 10 μM Q-VD-OPH for the indicated times and then cultured for additional 7 days. * p<0.05 vs control, ** p<0.01 vs control.

To further investigate the role of caspase activation in cardiomyocyte derivation from EBs, Q-VD-OPH was added to the differentiating EB cultures. The cultures were treated for 5 h, 24 h or 48 h and the expression levels of progenitor and cardiomyocyte markers were determined after 7 days. We found that α-MHC (Fig. 5A) and Nkx 2.5 (Fig. 5B) levels were significantly decreased by acute Q-VD-OPH treatment (5h), while chronic treatment (24 h and 48 h) only affected α-MHC expression. In contrast, Islet-1 expression (Fig. 5C) was strongly up-regulated by acute Q-VD treatment. This is in concordance with previous reports showing that caspase inhibition leads to accumulation of β-catenin, which in turn has been demonstrated to regulate this cell population [24,25]. Islet1 expression was not affected by chronic STS treatment. On the other hand, c-Kit expression (Fig. 5D) remained unchanged after acute Q-VD-OPH treatment and tended to decrease after chronic treatment. The pluripotency marker Oct-4 was also significantly increased in the presence of the caspase inhibitor (Fig. 5E), which is in agreement with the potent reduction of STS-induced caspase activity whereby Q-VD (OMe)-OPH reversed the STS-induced caspase activity down to levels below the untreated control (S1C Fig.), suggesting a role for caspase in the differentiation of the EBs. These results suggest that caspase activation acts on the cardiac progenitor cell population.

## Discussion

**Fig 6:**
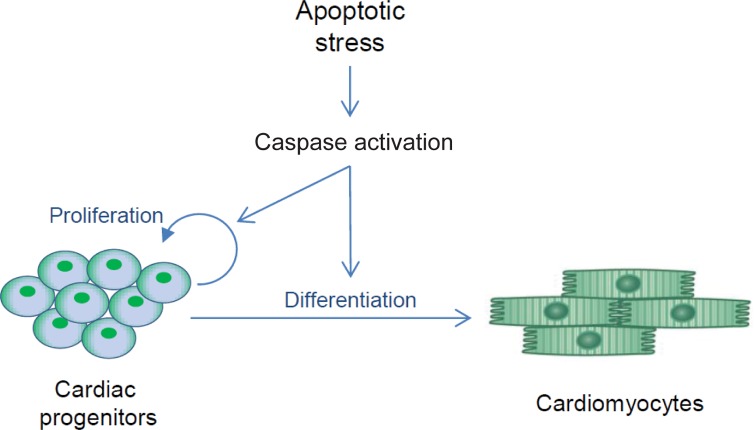
Proposed model. Exposure of progenitor cells to a sublethal apoptotic stress leads to caspase activation below the threshold necessary to cause apoptotic cell death. Activation of caspases leads to cardiac progenitor cells response characterized by expansion of existing progenitor cells and promotion of cardiogenic differentiation.

In this study we describe that sublethal pro-apoptotic stimuli leading to caspase activation play a significant role in embryonic stem cell differentiation towards cardiomyocytes. Our data indicates that caspase activation mainly affects the fate of cardiac progenitor cells by stimulating self-renewal and differentiation into cardiomyocytes, which is schematically described in Fig. 6.

Caspase activity has usually been associated with cell death. However, an increasing body of evidence suggests that this protease family also mediates cell fate decisions distinct from cell survival or death [11,12]. For instance, Drosophila spermatid differentiation [26] is tightly regulated through ubiquitin-mediated degradation of caspase inhibitory proteins [27], ensuring that the caspase activity guides differentiation rather than initiating the apoptotic program. In other systems, transient caspase 3 activation is required for the differentiation of various cell types, including neural precursor cells, hematopoietic cells and skeletal myoblasts [12,16,17,18,28]. In mESC cells, inhibition of caspases 3 and 8 impairs cardiomyocyte differentiation [29,30], while doxycycline-mediated activation of caspase 3 stimulates differentiation and at the same time decreases the expression levels of Nanog and Oct-4 [15]. A recent report by Akbari-Birgani *et al*. suggests that delay in the release of cytochrome *c*, apoptosome formation and caspase 3 activation discriminates apoptosis from differentiation in mESCs [31]. However, from these studies it is not known which cell population is affected by the caspase activation or inhibition.

Our results suggest that activation of caspases specifically affects the population of c-Kit^+^/α-actinin^low^ cells by stimulating their proliferation and differentiation into cardiomyocytes. According to our findings, sublethal STS stimuli lead to an increase in caspase 9 and executor caspase 3 activities, without causing a significant decrease in the mitochondrial membrane potential. This is in concordance with Murray *et al*. and their findings regarding a non-apoptotic role for caspase 9 in muscle differentiation [32]. The sublethal apoptotic stimuli mainly affected the population of more immature cells, which stained weakly for α-actinin and had a round morphology co-localized with c-Kit expression (c-Kit^+^/α-actinin^low^ cells). These cells formed clusters in close proximity to cells that stained strongly for α-actinin and had a more mature phenotype (α-actinin^high^ cells). Sublethal apoptotic stimuli also increased incorporation of EdU in the c-Kit^+^/α-actinin^low^ cells, without affecting the population of mature α-actinin^high^ cells. Furthermore, following STS treatment we also detected a significant increase of GFP expression in single cells, which is under control of the α-myosin heavy chain (α-MHC) promoter, suggesting increased differentiation of early cardiomyocytes. Beating clusters of cells with low GFP expression were also observed following STS exposure, but not in the untreated control cells. The addition of the caspase inhibitor Q-VD-OPH blocked these effects.

These findings might in part explain the up-regulation of endogenous c-Kit^+^ cardiac progenitor cells and their differentiation into cardiomyocytes following diffuse acute myocardial damage previously described by Ellison *et al*.[8]. In their study, c-Kit^+^ cardiac progenitor cells entered the cell cycle followed by commitment to the myocyte lineage, which was further supported by their co-expression of Nkx 2.5 and α-actinin. The generated cardiomyocytes expressed sarcomeric contractile proteins and at the same time maintained the gene expression typical of immature, not yet terminally differentiated cardiomyocytes. In other reports, the c-Kit^+^ cell population was transiently up-regulated after ischemia-reperfusion injury and myocardial infarction [5,23,33]. In another study by Senyo *et al*. [2], the authors demonstrated DNA replication in cells residing in the infarction border zone. As these cells displayed activated myosin heavy chain, the DNA replication was interpreted as division of pre-existing post-mitotic myocytes. However, in light of the present study, this finding might instead reflect proliferation of committed progenitor cells. Altogether, this means that our findings are in accordance with those previously presented, and furthermore, we highlight the possible mechanisms by which stem cells with cardiac differentiation potential may become activated following injury.

Our observation that caspase inhibition prevented up-regulation of cardiac-specific markers further confirms the role of caspases in cardiac differentiation, similarly to what has been described for skeletal muscle differentiation [18]. It has also been proposed that apoptosis and muscle differentiation induce similar cytoskeletal and phenotypical changes and thus share activation of similar molecular pathways [18,32]. A mechanism of action by which active caspase 3 induces cardiac differentiation might be through cleavage and activation of pro-differentiation, mitogen-activated protein kinase (MAPK) signaling cascades (MST1/MKK6/p38) [18]. Downstream components of these cascades up-regulate MEF2A and C factors known to be important for cardiomyocyte differentiation [34]. In the work by Abdul-Ghani *et al*. [29], the authors demonstrate that caspases have a role in cardiac differentiation mediated through their proteolytic cleavage of β-catenin, leading to a loss of canonical Wnt signaling activity [25]. Furthermore, actin fiber disassembly and reorganization are conserved features of both apoptosis and myoblast differentiation. The observation that the use of an apoptosis repressor reduces differentiation of H9c2 myoblasts [30] is in agreement with our observation that the caspase inhibitor decreases cardiomyocyte differentiation. Caspase 3-dependent signaling involved in cardiac differentiation could also be initiated by the activation of the caspase 8 pathway. In our study, caspase 8 was also activated, and this activation is most probably mediated through the STS induced recruitment of FADD [22]. However, 100 nM STS did not activate caspase 10, which in turn is processed by caspase 8 leading to cleavage and activation of caspase 3.

The FAS/FAS ligand system activates apoptosis in many cell types, although Badorff *et al*. [35] showed that cardiomyocyte-specific downstream signaling of the FAS receptor leads to survival and hypertrophy instead of apoptotic cell death [35]. In their study, FAS ligand increased protein synthesis, cell size and sarcomeric organization in neonatal cardiomyocytes and lead to up-regulation of atrial natriuretic factor (ANF), a hallmark of cardiac hypertrophy [35]. We did not find any evidence for increased hypertrophy in STS-treated EBs and this might be a reflection of the milder pro-apoptotic stimuli used in our study.

## Conclusion

In conclusion, we describe for the first time that a sublethal apoptotic stimulus triggers a caspase-dependent signaling mechanism, which controls the fate decisions of cardiac progenitor cells. Our results suggest that caspase activation promotes differentiation of c-Kit^+^/α-actinin^low^ cardiac progenitor cells, whereas caspase inhibition decreases cardiomyogenesis. In clinically relevant settings, these data may suggest that caspase-controlled pathways play a role in bridging an acute cell injury to the determination of cell fate in cardiac progenitors.

## Supporting Information

S1 Fig STS induces dose-dependent activation of caspases in differentiating EBs. (A) Titration of STS concentration upon plasma membrane permeabilization (Hoechst/PI nuclear counterstaining in live cultures). (B) Enzymatic caspase 3 activity in response to stimulation with increasing concentrations of STS for 5 h. (C) Enzymatic caspase 3 activity in EBs stimulated with 100 nM STS for 5 h in the presence of increasing concentrations of broad spectrum caspase inhibitor Q-VD-OPH. (D and E) Enzymatic caspases 8 and 10 activity in response to stimulation with 1μM STS, 100 nM STS +/- Q-VD-OPH and 10 μM Q-VD-OPH for 5 h. Bars represent100 μm. *p<0.05 vs control, **p<0.01 vs control, ##p<0.01 vs STS 100 nM, ###p<0.001 vs STS 100 nM.
(TIF)

S2 Fig Up-regulation of c-Kit after sublethal caspase activation. (A) EBs were treated for 5 h with 100 nM STS +/- Q-VD-OPH in the beginning of differentiation and subsequently cultured for three weeks. Protein expression of c-Kit is related to β-actin; two representative samples in each group are shown. (B) Expression of α-actinin (green), c-Kit (red) and EdU (far red) in differentiated EBs exposed to 100 nM STS +/- Q-VD-OPH for 5 h and then cultured for additional 7 days. Nuclei stain blue by DAPI. Bars represent 200 μm.
(TIF)

S3 Fig Expression of cardiomyocyte hypertrophy markers in EBs. Relative mRNA levels of hypertrophy markers β-MHC (A) and ANP (B) in EBs exposed to control vehicle, 100 nM STS or 100 nM STS + 10 μM Q-VD-OPH for 5 h and then collected for analysis after 7 days.
(TIF)
