# Supplementary figures and images for: Sublethal Caspase Activation Promotes Generation of Cardiomyocytes from Embryonic Stem Cells
Source: PLoS One. 2015 Mar 12;10(3):e0120176. doi: 10.1371/journal.pone.0120176 (PMC4357377; doi:10.1371/journal.pone.0120176)

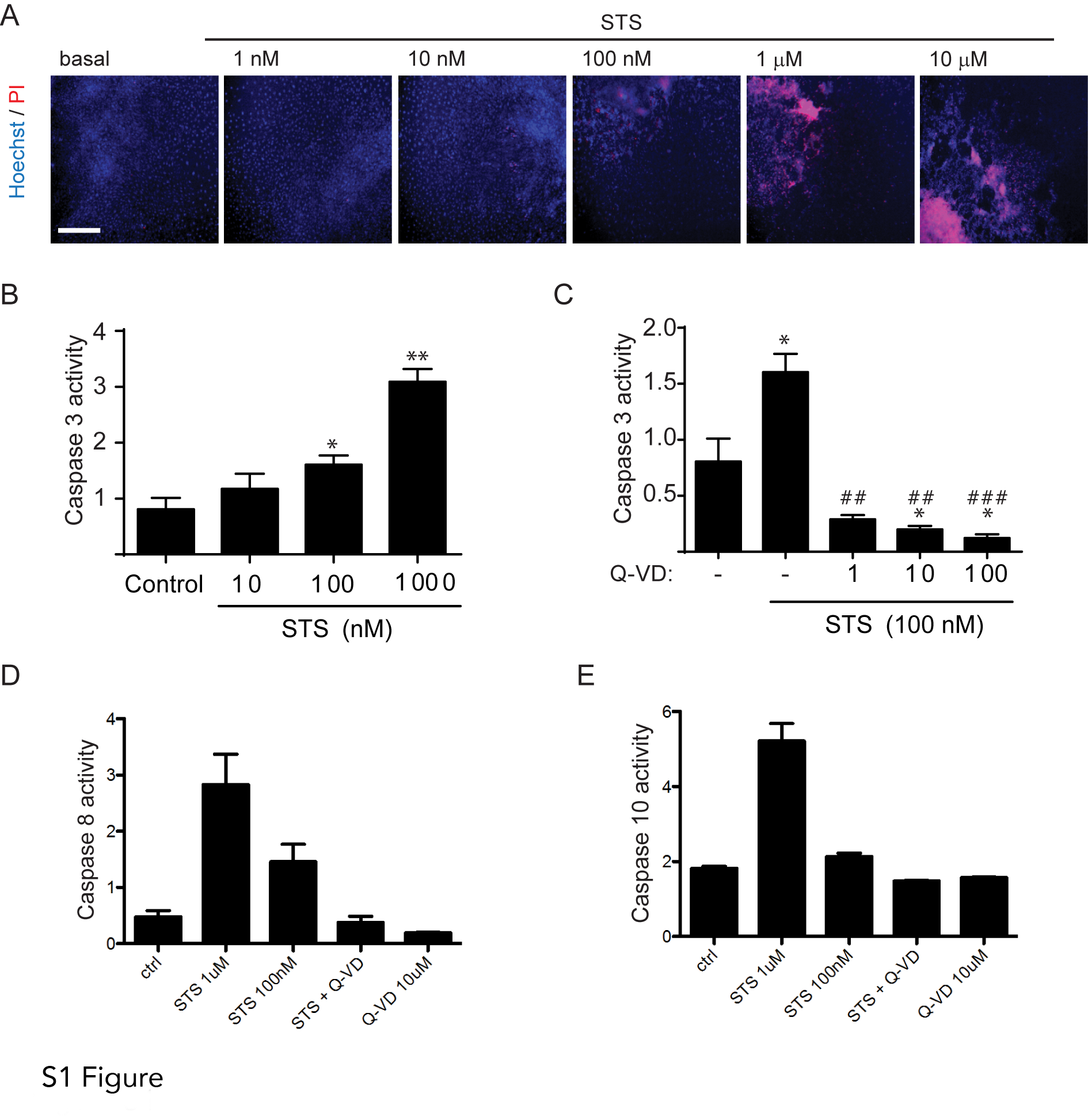

Supplement: S1 Fig — (A) Titration of STS concentration upon plasma membrane permeabilization (Hoechst/PI nuclear counterstaining in live cultures). (B) Enzymatic caspase 3 activity in response to stimulation with increasing concentrations of STS for 5 h. (C) Enzymatic caspase 3 activity in EBs stimulated with 100 nM STS for 5 h in the presence of increasing concentrations of broad spectrum caspase inhibitor Q-VD-OPH. (D and E) Enzymatic caspases 8 and 10 activity in response to stimulation with 1μM STS, 100 nM STS +/- Q-VD-OPH and 10 μM Q-VD-OPH for 5 h. Bars represent100 μm. *p<0.05 vs control, **p<0.01 vs control, ##p<0.01 vs STS 100 nM, ###p<0.001 vs STS 100 nM. (TIF) [file pone.0120176.s001.tif]

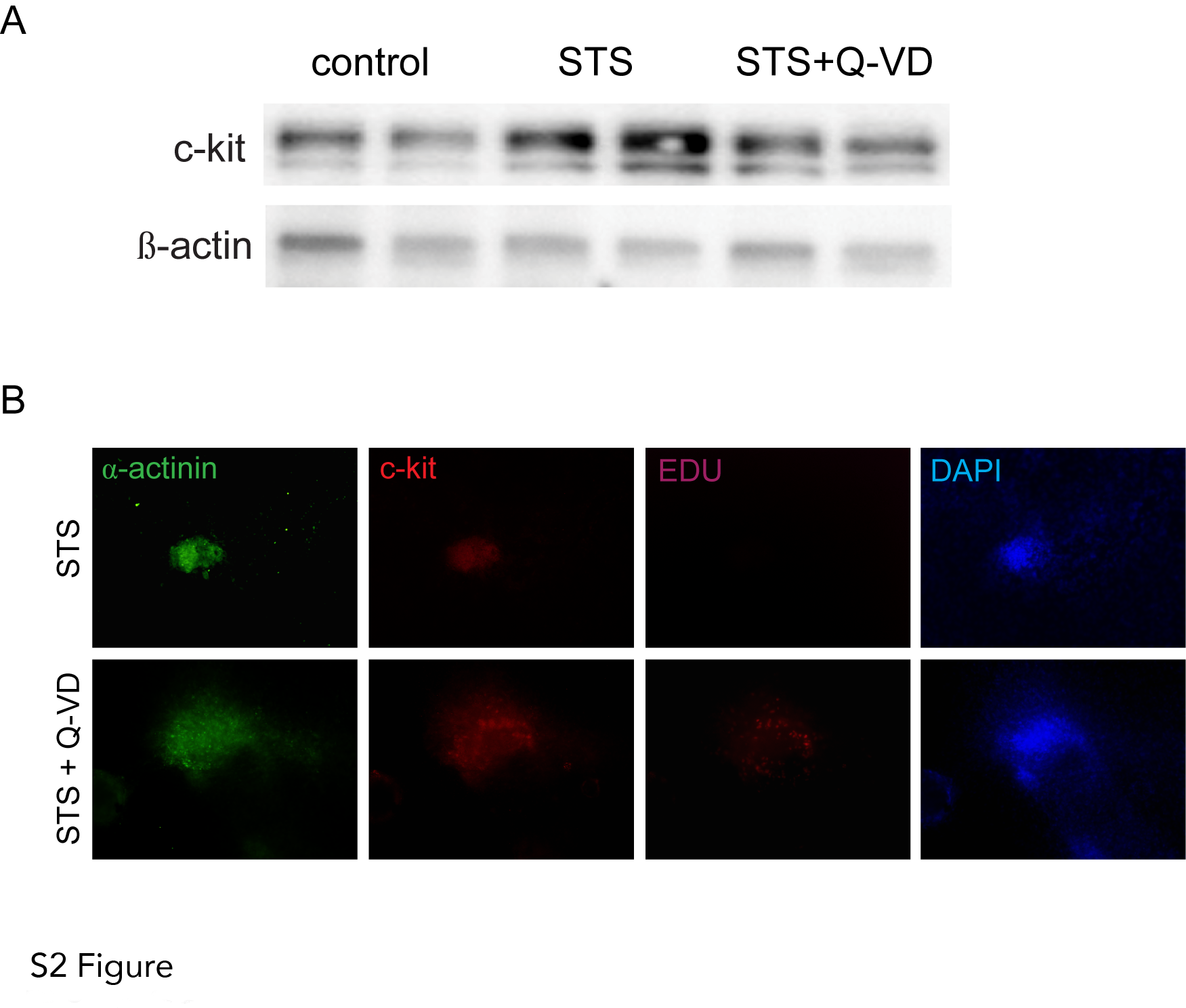

Supplement: S2 Fig — (A) EBs were treated for 5 h with 100 nM STS +/- Q-VD-OPH in the beginning of differentiation and subsequently cultured for three weeks. Protein expression of c-Kit is related to β-actin; two representative samples in each group are shown. (B) Expression of α-actinin (green), c-Kit (red) and EdU (far red) in differentiated EBs exposed to 100 nM STS +/- Q-VD-OPH for 5 h and then cultured for additional 7 days. Nuclei stain blue by DAPI. Bars represent 200 μm. (TIF) [file pone.0120176.s002.tif]

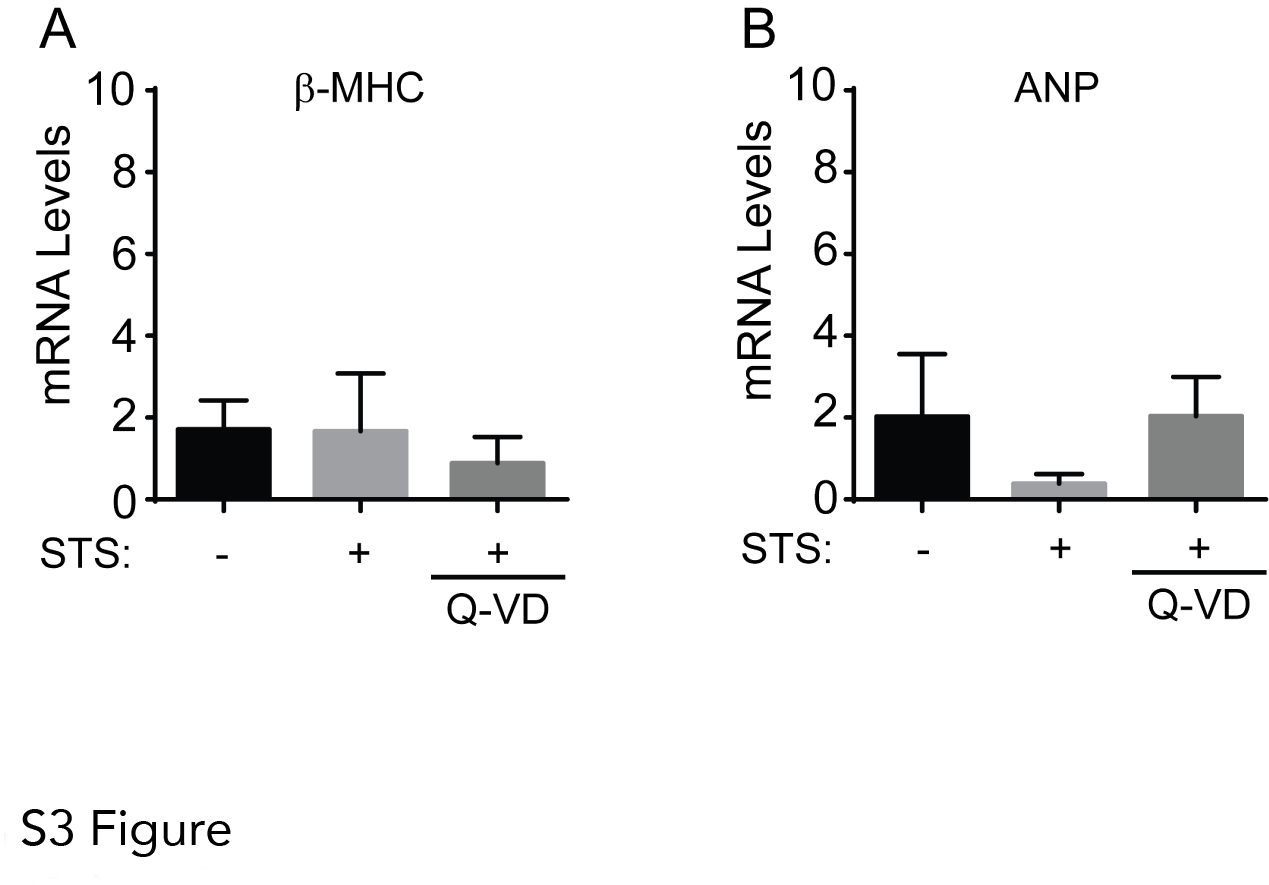

Supplement: S3 Fig — Relative mRNA levels of hypertrophy markers β-MHC (A) and ANP (B) in EBs exposed to control vehicle, 100 nM STS or 100 nM STS + 10 μM Q-VD-OPH for 5 h and then collected for analysis after 7 days. (TIF) [file pone.0120176.s003.tif]
